# Primary Cutaneous Diffuse Large B-cell Lymphoma Successfully Treated With R-CHOP Chemotherapy

**DOI:** 10.7759/cureus.55300

**Published:** 2024-03-01

**Authors:** Wafa M Alghamdi, Fawaz H Aljehani, Abdullah M Alharthi, Reda I Bakhsh

**Affiliations:** 1 Dermatology, King Abdulaziz Hospital, Makkah, SAU; 2 Medical Oncology, Al-Noor Specialist Hospital, Makkah, SAU

**Keywords:** r-chop chemotherapy, primary cutaneous diffuse large b-cell lymphoma, r-chop, primary, lymphoma, cutaneous, b-cell

## Abstract

Primary cutaneous diffuse large B-cell lymphomas (PCDLBCLs) represent approximately 10%-20% of primary cutaneous B-cell lymphomas. They present as nodules in the skin or as rapidly growing aggressive behavior tumors with a poor prognosis. In this article, we report a case of PCDLBCL presented with an aggressively enlarging skin lesion on the right cheek. This case was diagnosed based on clinicopathological features and characteristic immunohistochemical expression. During the 11-month follow-up period, the patient showed significant clinical improvement after undergoing rituximab plus cyclophosphamide, doxorubicin, vincristine, and prednisone, abbreviated as R-CHOP chemotherapy, without evidence of extracutaneous dissemination or disease relapse.

## Introduction

Primary cutaneous lymphomas (PCLs) are a heterogeneous group of lymphoproliferative disorders that present primarily as skin involvement without extracutaneous evidence at the time of diagnosis [1]. Moreover, PCLs show considerable variation in prognosis and treatment from systemic lymphomas with similar histological features. Primary cutaneous lymphomas are rare, with an incidence of 0.5 to one case per 100,000 people each year [2]. There are two types of cutaneous lymphomas. Most cases are cutaneous T-cell lymphomas (~71% of all cutaneous lymphomas). Cutaneous B-cell lymphomas (CBCL) are less common (29% of cases) [1].

According to the 2018 update of the World Health Organization-European Organization for Research and Treatment of Cancer (WHO-EORTC), CBCLs can be divided into four main categories that are as follows: primary cutaneous marginal zone B-cell lymphoma (PCMZL), primary cutaneous follicular center lymphoma (PCFCL), intravascular diffuse large B-cell cutaneous lymphoma (IVDLBCL), and primary cutaneous diffuse large B-cell lymphoma-leg type (PCDLBCL-LT) [3]. Epstein-Barr virus-positive mucocutaneous ulcer (EBV-MCU) is a new provisional entity listed as a WHO classification update in 2018 [3]. The first two categories, in addition to EBV-MCU, grow slowly, while the others are more aggressive subtypes associated with a high death rate [2]. In general, histopathological characteristics and immunohistochemistry analysis can help to distinguish between these subtypes. Here, we report a case of cutaneous diffuse large B-cell lymphoma on the face. The diagnosis was made clinicopathologically with the aid of dermoscopy. Histologically and clinically, the patient showed significant improvement after receiving chemotherapy.

## Case presentation

**Figure 1:**
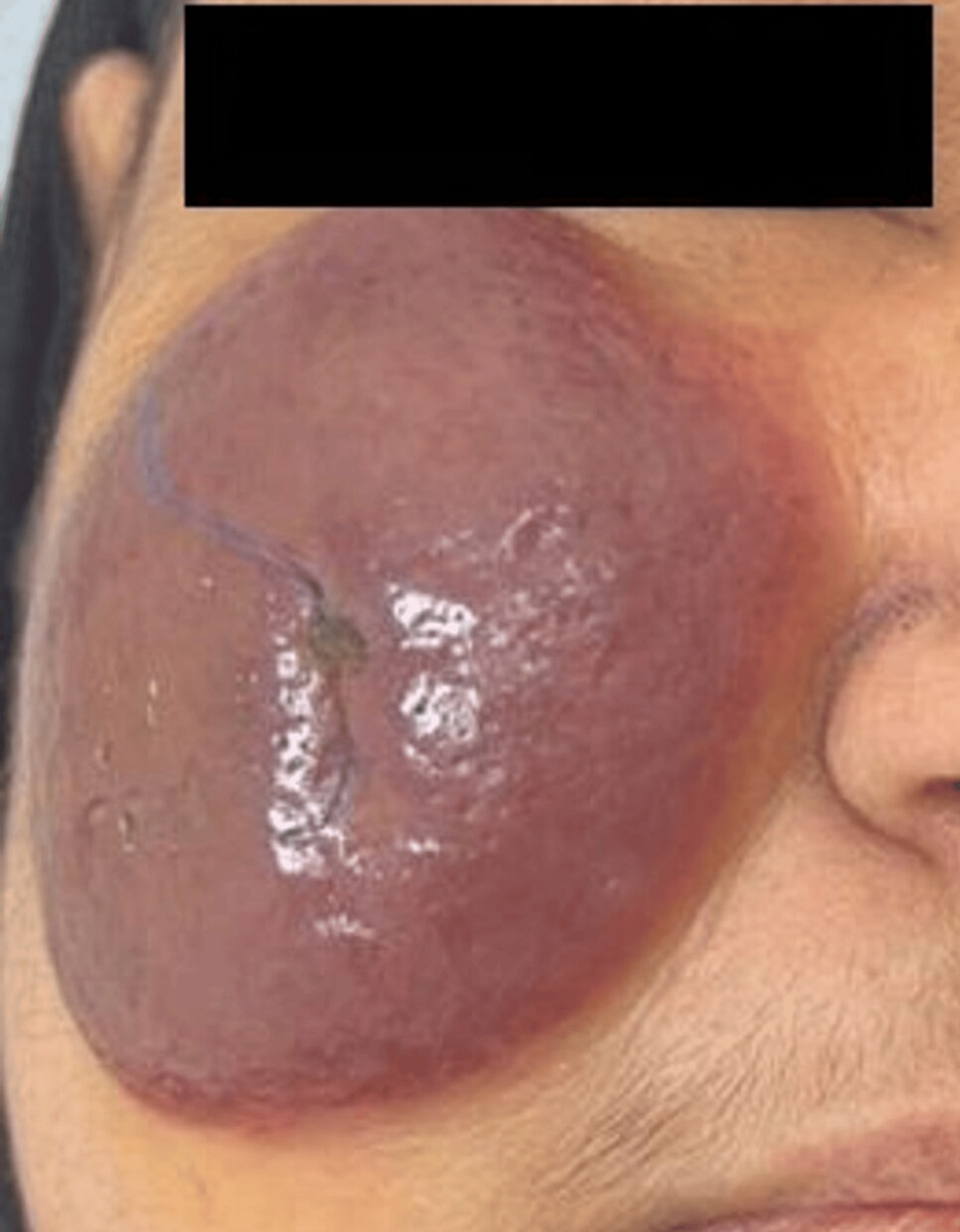
Clinical manifestations before chemotherapy A solitary, well-demarcated nodule with telangiectasia over the cheek is seen.

A 46-year-old woman with no known medical illness presented to our dermatology department for evaluation of an erythematous nodule over the right cheek that had evolved over the last three months, compressing her right eye. She denied pain, bleeding, or other local symptoms. No other body site was involved. The systematic review was unremarkable. There is no significant family history of skin disease or tumors. The physical examination showed a solitary, well-demarcated, shiny nodule that was reddish pink, measuring 4.2x6.0 cm, and had telangiectasia over the right cheek (Figure 1). 

**Figure 2:**
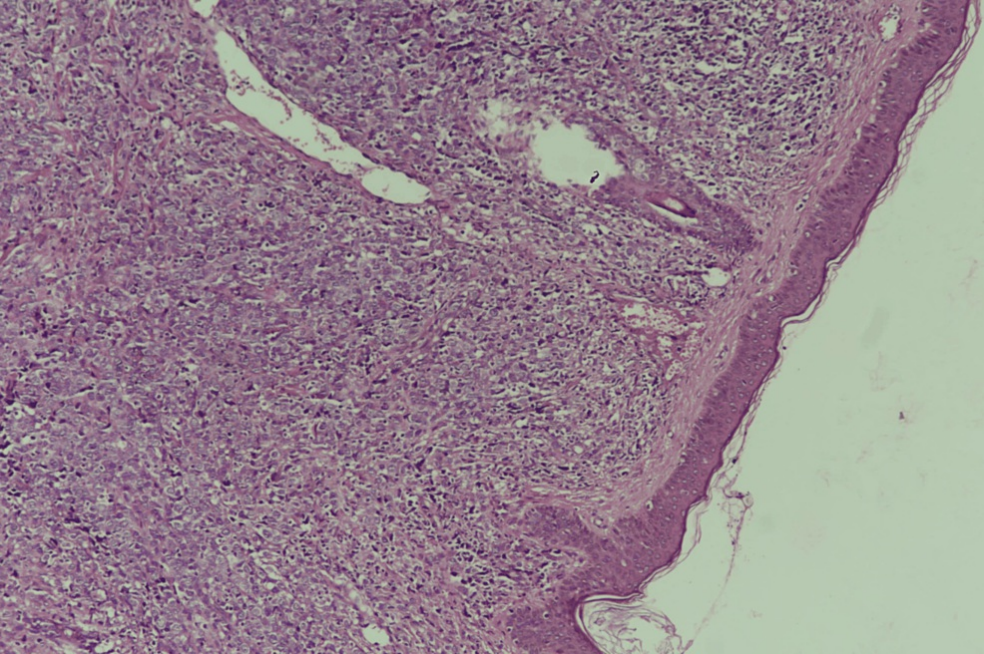
Histopathology image Low-power view shows malignant lymphoid cells infiltrating the dermis.

**Figure 3:**
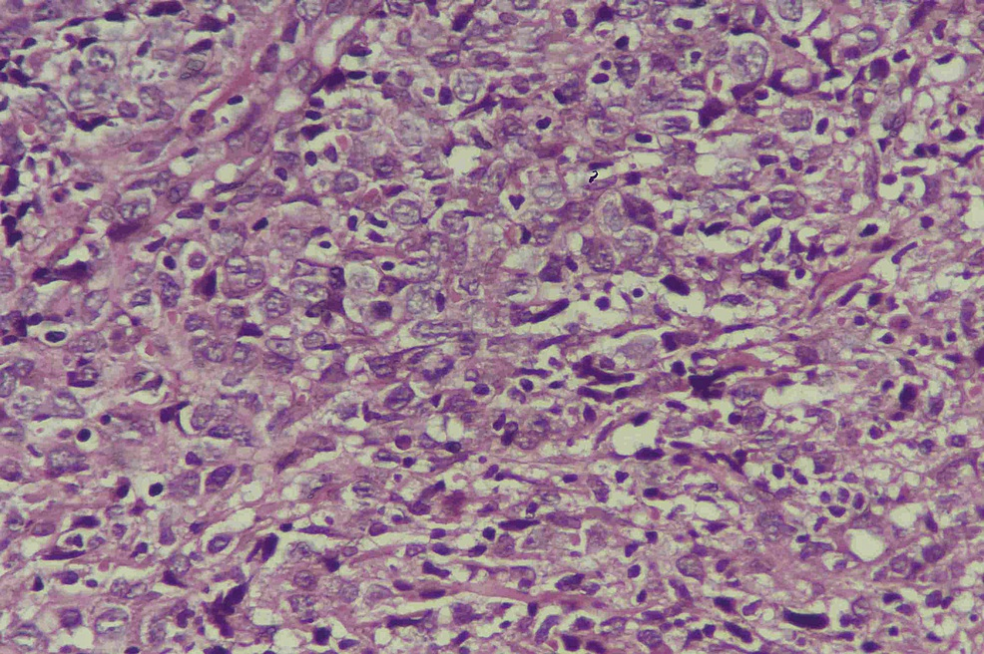
Histopathology image The high-power view shows the malignant lymphoid cells exhibiting pleomorphism, hyperchromasia, and frequent mitosis.

**Figure 4:**
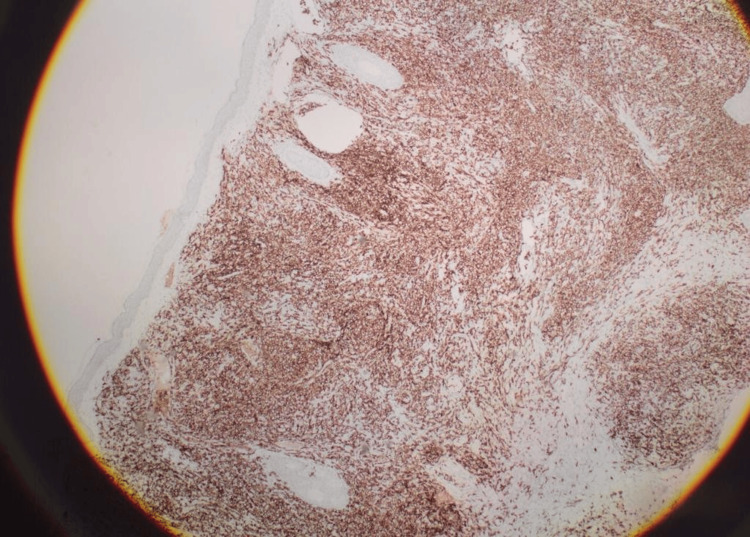
Positive results of leukocyte common antigen (LCA) The LCA immunostain shows diffuse positivity of the malignant cells.

**Figure 5:**
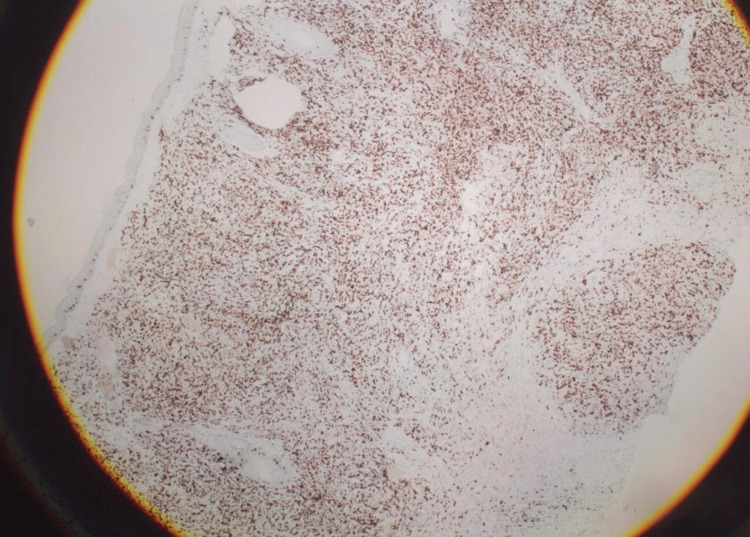
Positive results of CD79 CD79 (B-cell marker) shows diffuse positivity of the malignant cells.

**Figure 6:**
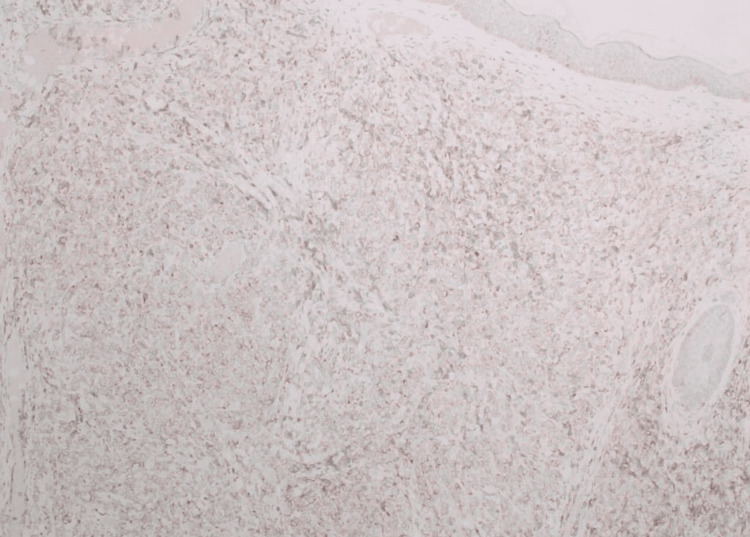
Positive results of the B-cell lymphoma 2 (BCL2) marker The BCL2 marker shows focal positivity of the malignant cells.

The lesion had a firm consistency and no tenderness. Otherwise, the skin examination was normal. No lymph nodes were palpable in the cervical, axillae, or clavicular regions. A cutaneous biopsy revealed diffuse dermal and subcutaneous infiltration of medium- to large hyperchromatic pleomorphic cells with no epidermal involvement by tumor cells. These cells were positively stained for leukocyte common antigen (LCA), CD79a, and B-cell lymphoma 2 (BCL2) (Figures 2-6). 

**Figure 7:**
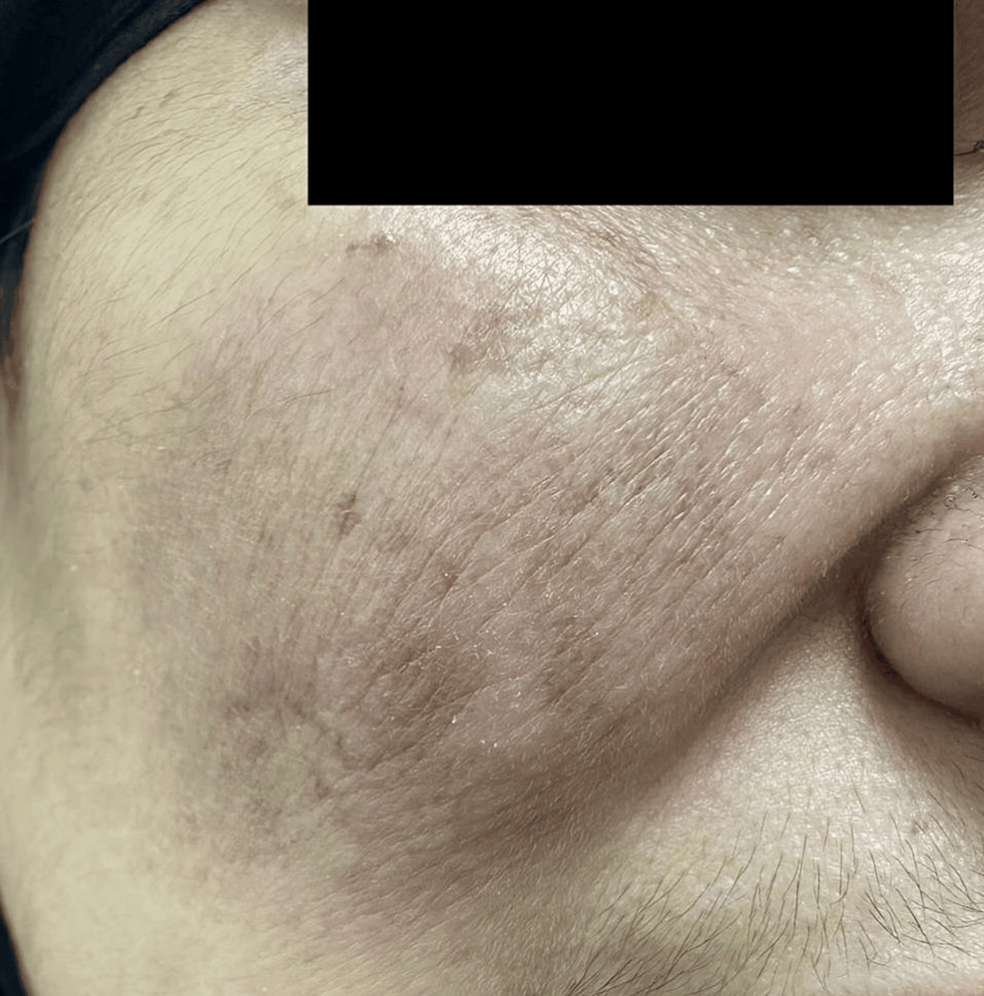
Clinical manifestations after chemotherapy The tumor appears to have significantly reduced and flattened.

The complete blood count, biochemical studies, and serology tests were normal. A CT scan found dermal and subdermal right cheek involvement with mild increasing vascularity and multiple variable-sized lymph node enlargement at the right submandibular region with no necrosis or calcification and normal CT appearance of the chest, abdomen, and pelvis. Therefore, based on clinicopathological features, a diagnosis of PCDLBCL was established. The case was later referred to the oncology team, and she started four cycles of rituximab plus cyclophosphamide, doxorubicin, vincristine, and prednisone (R-CHOP) chemotherapy every 21 days. There was almost complete resolution after the fourth cycle, with no side effects during chemotherapy (Figure 7).

## Discussion

Primary cutaneous diffuse large B-cell lymphoma is a subtype of CBCL and is defined as a lymphoma composed of large cells accounting for more than 80% of the infiltrate with no extracutaneous evidence at the time of diagnosis and after completion of an initial staging investigation. A PCDLBCL mostly occurs on the legs (PCDLBCL-LT), but rarely it can involve other cutaneous sites; extracutaneous dissemination is common [4]. The occurrence of PCDLBCL in other anatomic areas is rarely studied, and diagnosis can be challenging due to non-specific symptoms and signs. Thus, an accurate diagnosis requires a biopsy and immunohistochemical analysis. The presence of more than one skin lesion, round-cell morphology, and location on the leg are independent factors associated with a worse prognosis [5]. None of these were present in our patient.

The literature is still limited on therapeutic approaches. Primary cutaneous diffuse large B-cell lymphoma-leg type exhibits aggressive behavior. The first-line therapeutic intervention is systemic rituximab and combination chemotherapy, most commonly cyclophosphamide, doxorubicin, vincristine, and prednisone (CHOP)-like regimens. If there is no contraindication, then other options mentioned in the literature include local radiotherapy, along with excision, and pegylated liposomal doxorubicin [6]. There was also one case report of PCDLBCL-LT treated with rituximab and bendamustine. Unfortunately, the tumors progressed after two courses of treatment [7]. In another report, as PCDLBCL-LT has a mutation in myeloid differentiation factor 88 (MYD88-L265P), a combination of rituximab, lenalidomide, and ibrutinib (Bruton tyrosine kinase inhibitor) offered meaningful outcomes in two patients with recalcitrant PCDLBCL-LT [8].

## Conclusions

Our patient presented with an atypical clinical presentation of cutaneous B-cell lymphoma. Histopathological and immunohistochemical examinations revealed PCDLBCL-other (PCDLBCL-O). We used R-CHOP chemotherapy, which is widely recommended, and it showed a favorable response after four cycles. However, a close follow-up evaluation is still warranted to assess recurrence and long-term side effects. This case report will expand knowledge about this type of lymphoma so that more professionals may add it to their differential diagnoses list and thus improve the prognosis.
